# Supplementary material for: Cryogenic quantum computer control signal generation using high-electron-mobility transistors
Source: Commun Eng. 2024 Oct 15;3:146. doi: 10.1038/s44172-024-00293-2 (PMC11480393; doi:10.1038/s44172-024-00293-2)
Supplement: Supplementary file 2 — Supplementary Information [file 44172_2024_293_MOESM2_ESM.pdf]

Supplementary material

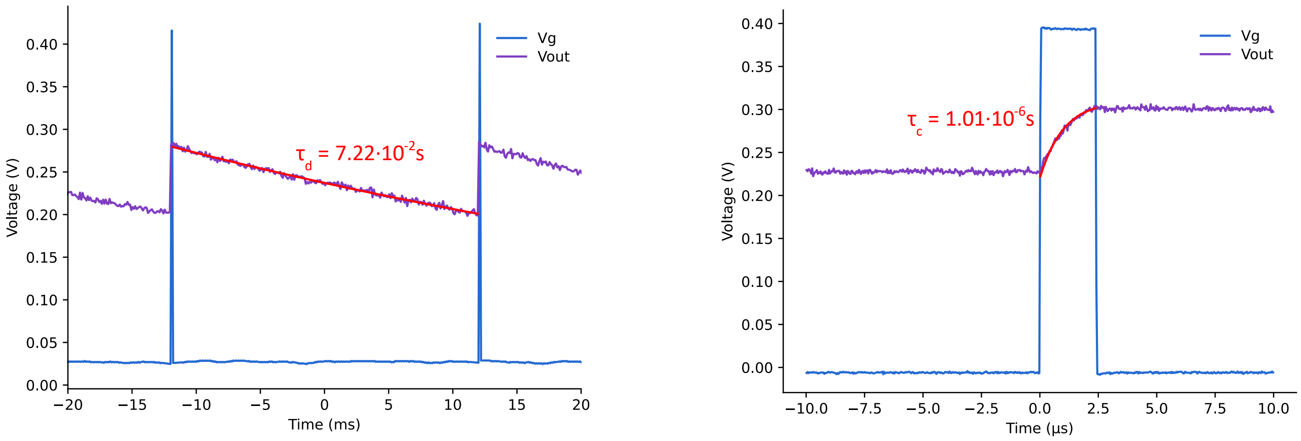

Supplementary Fig. S1: Extraction of the charge and discharge time constants by fitting the voltage measured on the capacitor with an exponential function.

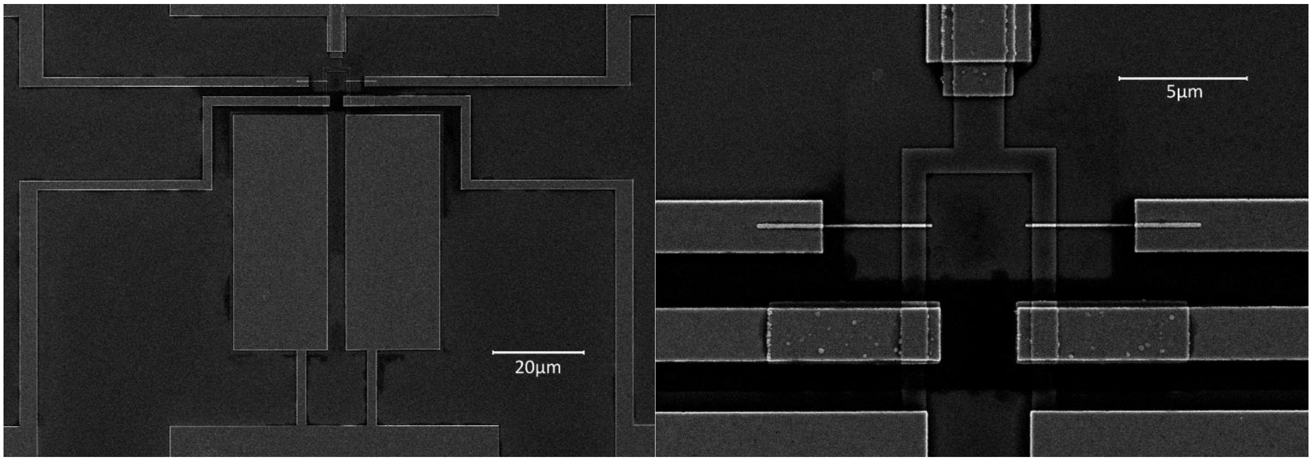

Supplementary Fig. S2: SEM images of the 2x1 array used to characterize the charge storage functionality and the crosstalk between two adjacent cells.
